# Quantum Misuse Attack on Frodo

**DOI:** 10.3390/e24101418

**Published:** 2022-10-04

**Authors:** Yaru Wang, Haodong Jiang, Zhi Ma

**Affiliations:** State Key Laboratory of Mathematical Engineering and Advanced Computing, Zhengzhou 450001, China

**Keywords:** learning with problem, lattice-based cryptography, quantum misuse attack, Frodo, quantum algorithm

## Abstract

Research on the security of lattice-based public-key encryption schemes against misuse attacks is an important part of the cryptographic assessment of the National Institute of Standards and Technology (NIST) post-quantum cryptography (PQC) standardization process. In particular, many NIST-PQC cryptosystems follow the same meta-cryptosystem. At EUROCRYPT 2019, B$\overset{˘}{a}$etu et al. mounted a classical key recovery under plaintext checking attacks (KR-PCA) and a quantum key recovery under chosen ciphertext attacks (KR-CCA). They analyzed the security of the weak version of nine submissions to NIST. In this paper, we focus on learning with error (LWE)-based FrodoPKE, whose IND-CPA security is tightly related to the hardness of plain LWE problems. We first review the meta-cryptosystem and quantum algorithm for solving quantum LWE problems. Then, we consider the case where the noise follows a discrete Gaussian distribution and recompute the success probability for quantum LWE by using Hoeffding bound. Finally, we give a quantum key recovery algorithm based on LWE under CCA attack and analyze the security of Frodo. Compared with the existing work of B$\overset{˘}{a}$etu et al., our method reduces the number of queries from $2^{2}$ to 1 with the same success probability.

## 1. Introduction

Quantum computing exploits quantum mechanical properties to perform computations. It enables quantum parallelism and provides much more powerful data processing capabilities than classical computers [1]. In 1994, Peter Shor proposed an efficient quantum algorithm [2] that can break most of the current public-key cryptosystems, such as the Diffie–Hellman protocol [3] and RSA cryptosystem [4]. If large-scale quantum computers are realized, they would threaten the security of many public-key cryptosystems. In order to ensure the security of network information systems, NIST initiated a standardization process for post-quantum algorithms. In 2016, NIST called for proposals for post-quantum cryptosystems [5]. There are 69 candidates in the first round, based on a variety of hard problems considered to be intractable by quantum computers. After rigorous scrutiny by the cryptography community, 17 PKE and key encapsulation mechanisms (KEM) candidates were selected in the second round, where nine are lattice-based. In the third round, three of the four finalists are still lattice-based. In 2022, NIST has completed the third round of the PQC standardization process. A total of four candidate algorithms have been selected for standardization, and four additional algorithms will continue into the fourth round. The selected algorithms are mostly lattice-based cryptography [6]. Lattice-based cryptography is the use of conjectured hard problems on point lattices in $R^{n}$ as the foundation for secure cryptographic systems. Attractive features of lattice cryptography include apparent resistance to quantum attacks, high asymptotic efficiency and parallelism, security under worst-case intractability assumptions, and solutions to long-standing open problems in cryptography. Lattice cryptography has some attractive features, including (1) conjectured security against quantum attacks, (2) algorithmic simplicity, efficiency, and parallelism, (3) strong security guarantees from worst-case hardness, and (4) constructions of versatile and powerful cryptographic objects.

In general, most lattice-based NIST-chosen plaintext attack (CPA) secure candidates use the Fujisaki–Okamoto (FO) transformation [7] to achieve IND-CCA security. When the key is reused, the CPA-secure PKE is no security guarantee. Research on key reuse attacks against lattice-based CPA-secure schemes is an important topic in the post-quantum cryptography. Many key-recovery attacks have been proposed in [8,9,10,11,12,13]. In 1998, Bleichenbacher showed the security of IND-CPA secure public-key cryptosystems in the case of key reuse on RSA encryption standard PKCS#1 [14]. In 2010, Menezes et al. gave the key reuse attack on reusing ephemeral keys in Diffie–Hellman key agreement protocols [15]. In 2016, Fluhrer proposed a key reuse attack [16]. In 2017, Ding et al. expanded Fluhrer’s attack to a class of key agreement protocols based on ring-LWE with signaling [17]. In 2019, Bauer et al. [18] gave a key-recovery attack on NewHope-CPA-PKE [19]. In 2021, Yue Qin et al. developed a systematic approach and analyzed key misuse attacks on lattice-based NIST candidates [20]. Although there have been a number of classical key misuse attacks on the lattice-based public key encryption schemes, quantum misuse attack algorithms are rarely studied. In 2019, Alagic et al. gave a quantum algorithm for learning rounding function and showed that this algorithm can recover the key of an IND-CPA-secure LWE-based encryption scheme with constant success probability [21]. At EUROCRYPT 2019, B$\overset{˘}{a}$etu et al. analyzed the security of meta-cryptosystems under key reuse by mounting a quantum key recovery under the chosen-ciphertext attacks [22].

Although NIST did not select Frodo as the initial post-quantum algorithm in the process of post-quantum cryptography standardization, Frodo remains a post-quantum recommendation of Germany’s Bundesamt für Sicherheit in der Informationstechnik (BSI) [23]. The FrodoPKE scheme is an instantiation and implementation of the Lindner–Peikert scheme [24] with some modifications, for example, more balanced key and ciphertext sizes and new LWE parameters. The IND-CPA security of FrodoPKE is tightly related to the hardness of a corresponding learning with errors problem. In 2005, Regev [25] defined the LWE problem, proved the hardness of LWE assuming the hardness of various worst-case lattice problems against quantum algorithms, and defined a PKE scheme whose IND-CPA security is based on the hardness of LWE. The LWE problem is a generalization of the learning parity with a noise problem [26] into large moduli *q*.

In this paper, we give an improved quantum algorithm for recovering the key of IND-CPA version of Frodo by using a quantum CCA attack. The security of Frodo’s proposal is based on a plain LWE problem. In lattice-based cryptography, the plain LWE problem [25] is to solve a noisy linear system modulo as a known integer.

The main contributions of this paper are as follows:

(1) Based on the improved quantum algorithm for solving the quantum LWE problem, we first recalculate the success probability when the error follows a discrete Gaussian distribution. Using Hoeffding bound, we give the success probability for solving quantum LWE by computing the expectation and variance of the error.

(2) Then, we present a quantum KR-CCA attack which is inspired by the quantum LWE solving algorithm. Based on the existing quantum LWE solving algorithm, we recompute the success probability by using a different method. We analyze the security of Frodo640, Frodo976 and Frodo1344. By computing the expectation and variance of the error term, we can recover the full key with fewer oracle queries. Compared with the work of B$\overset{˘}{a}$etu et al. [22], our algorithm can reduce the number of oracle calls to 1 and meanwhile keep the same success probability as the AJOP-based quantum KR-CCA algorithm; see Table 1.

The organization of our paper is as follows. In Section 2, we give basic definitions and the meta-cryptosystem defined in the algorithm. In Section 3, we review the quantum algorithm for solving quantum LWE. Then, we recalculate the success probability for solving quantum LWE problems when the noise follows a discrete Gaussian distribution. In Section 4, we propose an improved quantum key-recovery attack on LWE-based IND-CPA schemes and analyze the security of Frodo. We conclude the paper in Section 5. In addition, we give a table with the acronyms and their meaning in Abbreviations.

## 2. Preliminaries

### 2.1. Notation and Definitions

For an integer q≥1, let $Z_{q}$ be the residue class group modulo *q* such that $Z_{q}=\left\{0,1,\cdots ,q-1\right\}$. Let $x\overset{}{\to }X$ denote an element *x* is chosen according to uniform distribution from a finite set *X*. $x\overset{\chi }{\to }X$ denotes an element *x* is chosen according to χ distribution from a finite set *X*. For a random variable *y*, E[y] denotes the expectation value of *y*, Var[y] denotes the variance of *y*. Given a matrix *A*, $A^{T}$ will denote the transpose of *A*.

**Definition 1**  ((LWE) [25])**.** *Let n,q be positive integers, χ be a probability distribution on Z and s be a secret element in $Z_{q}^{n}$. We denote by L the probability distribution on $Z_{q}^{n}\times Z_{q}$ obtained by choosing $a\in Z_{q}^{n}$ uniformly at random, choosing $e\in Z_{q}$ by sampling each of its coefficients according to χ, and returning $\left(a,b\right)=\left(a,a\cdot s+e\right)\in Z_{q}^{n}\times Z_{q}$. Decision-LWE is the problem of deciding whether pairs $\left(a,b\right)\in Z_{q}^{n}\times Z_{q}$ are sampled according to L or the uniform distribution on $Z_{q}^{n}\times Z_{q}$. Search-LWE is the problem of recovering s from $\left(a,b\right)=\left(a,a\cdot s+e\right)\in Z_{q}^{n}\times Z_{q}$ sampled according to L.*

**Definition 2**  ((Quantum LWE) [27])**.** *The samples are given in the form of a uniform quantum superposition state $\frac{1}{\sqrt{q^{n}}}\sum_{a\in Z_{q}^{n}}|a\rangle |a\cdot s+e_{a}\left(\mathrm{mod}\;q\right)\rangle$ by querying a quantum oracle, where $e_{a}$ are independent identical distribution random variables from some distribution χ. The goal is to output s.*

**Definition** **3** (Public key encryption)**.** *A public key encryption scheme is a triple of randomized algorithms as follows:*

*(1) The key generator: given the security parameter, it outputs a public key and secret key.*

*(2) The encryption algorithm: takes a public key and a message (from some known set of valid messages) and outputs a ciphertext.*

*(3) The decryption algorithm takes a secret key and a ciphertext and outputs either a message or a distinguished “failure” symbol.*

*The scheme is said to be correct if generating a key pair, then encrypting a valid message using the public key, and then decrypting the resulting ciphertext using the secret key yields the original message (perhaps with all but negligible probability).*

**Definition** **4** (Quantum Fourier transform)**.** *For any positive integer q, the quantum Fourier transform over $Z_{q}$ is defined by the operation*

(1) $$QFT_{Z_{q}}|x\rangle =\frac{1}{\sqrt{q}}\sum_{y\in Z_{q}}\omega_{q}^{x\cdot y}|y\rangle$$

*where $\omega_{q}=e^{\frac{2\pi i}{q}}$.*

**Definition** **5** (Hoeffding’s bound). *Consider a set of k independent random variables $X_{i}$, such that $a_{i}\leq X_{i}\leq b_{i}$. Let $c_{i}=b_{i}-a_{i}$, $X=\sum_{i\in [n]}X_{i}$. The expectation value of X is μ=E[X]. Then, it follows that for any δ>0,*

(2) $$Pr\left[X-\mu \leq -\delta n\right]\leq e^{\frac{-2n^{2}\delta^{2}}{{\left(b_{i}-a_{i}\right)}^{2}}}$$

### 2.2. The Meta-Cryptosystem Defined on the Algebra

The meta-cryptosystem defined on the algebra was given by Băetu et al. [22] in 2019. Băetu et al. considered six additive Abelian groups $S_{\mathrm{sk}},S_{A},S_{B},S_{t},S_{U},S_{V}$ and its four bilinear mappings: $S_{A}\times S_{\mathrm{sk}}\to S_{B}$, $S_{U}\times S_{\mathrm{sk}}\to S_{V}$, $S_{t}\times S_{A}\to S_{U}$, $S_{t}\times S_{B}\to S_{V}$. The operation satisfies the associative law for bilinear mappings ×, that is (t×A)×sk=t×(A×sk) for all $t\in S_{t},A\in S_{A},sk\in S_{\mathrm{sk}}$.

For any plaintext pt∈M, we first define two functions: encode function $M\to S_{V}$ and decode function $S_{V}\to M$ such that encode function is injective. As shown in Table 2, we have

(3) $$\begin{array}{cc} \; & W=V-U\times sk\\ \; & =t\times B+f+encode(pt)-t\times A\times sk-e\times sk\\ \; & =t\times (A\times sk)+t\times d+f+encode(pt)-t\times A\times sk-e\times sk\\ \; & =t\times d-e\times sk+f+encode(pt), \end{array}$$

then W=δ+encode(pt) with δ=t×d−e×sk+f, where δ denotes the error introduced by encoding/decoding.

In fact, in many cryptosystems, the encode and decode functions are different. In particular, we give the encode and decode functions on Frodo in Section 4.2.

## 3. New Method for Solving Quantum LWE Problem

### 3.1. Quantum Algorithm for Solving Quantum LWE Problem

In 2019, Grilo et al. gave an efficient quantum-solving algorithm for the quantum LWE problem [28]. After, Wang et al. presented an improved quantum algorithm [27] based on the work of Grilo et al. In their algorithm, the noise $e_{u}$ is a random variable with the absolute value at most *k*. In the following, we first give the algorithm of Wang et al. Then, we consider the case where the noise follows a discrete Gaussian distribution and propose a new method of computing the success probability.

**Lemma** **1** ([27]). *Let $u,\mathrm{sk}\in Z_{q}^{n}$, $e_{u}\in \left[-k,k\right]$, $k<\frac{q}{4}$, q be subexponential in the dimension n. The algorithm can recover the secret key sk with the probability of at least $\frac{1}{q^{2n}}\left|\right|\sum_{u\in Z_{q}^{n}}\cos\frac{2\pi e_{u}}{q}{\left|\right|}^{2}$.*

From the algorithm process in Algorithm 1, the probability of outputting the key sk is

(4) Pr[sk]=1q2n||∑u∈Zqnω−eu||2=1q2n[(∑u∈ZqnRe(ω−eu))2+(∑u∈ZqnIm(ω−eu))2]≥1q2n(∑u∈ZqnRe(ω−eu))2=1q2n||∑u∈Zqncos2πeuq||2

Since $E(\sum_{u\in Z_{q}^{n}}\sin\frac{-2\pi e_{u}}{q})\to 0$, the first inequality holds.
**Algorithm 1:** Improved quantum algorithm for solving the quantum LWE problem.Quantum oracle: $|u\rangle |y\rangle \to |u\rangle |u\cdot \mathrm{sk}+e_{u}+y\rangle$1: Set the initial state to ${|0\rangle }^{⊗n}|1\rangle$2: Apply a quantum Fourier transform on the all registers   and obtain $\frac{1}{\sqrt{q^{n}}}\sum_{u\in Z_{q}^{n}}|u\rangle \frac{1}{\sqrt{q}}\sum_{x\in Z_{q}}\omega^{x}|x\rangle$3: Apply a quantum oracle query and obtain   $\frac{1}{\sqrt{q^{n}}}\sum_{u\in Z_{q}^{n}}\omega^{-u\cdot \mathrm{sk}-e_{u}}|u\rangle \frac{1}{\sqrt{q}}\sum_{x\in Z_{q}}\omega^{x}|x\rangle$4: Apply a quantum Fourier transform on the first register   and obtain $\frac{1}{q^{n}}\sum_{u,y\in Z_{q}^{n}}\omega^{-e_{u}}|y\rangle \frac{1}{\sqrt{q}}\sum_{x\in Z_{q}}\omega^{x}|x\rangle$5: Discard the second register and measure the first register6: Output sk

### 3.2. New Method

As shown in Equation (4), Wang et al. can obtain the success probability for solving the quantum LWE problem by using the method of enlarging and reducing, where the error $e_{u}\in \left[-k,k\right]$. In some lattice-based cryptosystems, the noise follows a discrete Gaussian distribution, such as Frodo. In this subsection, we recompute the success probability that the noise follows a discrete Gaussian distribution. The new method is explained as follows: by using Hoeffding bound in Equation (4), we can obtain the success probability with expectation value and variance. Then, we consider the case where the error $e_{u}$ follows the discrete Gaussian distribution and compute the expectation value and variance of $e_{u}$. The details are listed as follows.

Let $e_{u}$ follow the discrete Gaussian distribution $N(0,\sigma^{2})$, $e_{u}\in \left[-\frac{q}{2},\frac{q}{2}\right]$. The expectation of $e_{u}$ is $E(e_{u})=0$, the variance of $e_{u}$ is $\mathrm{Var}\left(e_{u}\right)=\sigma^{2}$, then $E\left(e_{u}^{2}\right)=E^{2}\left(e_{u}\right)+\mathrm{Var}\left(e_{u}\right)=\sigma^{2}$.

Using the mathematical analysis method, we first give the Taylor expansion of cosα

(5) $$\cos\alpha =1-\frac{\alpha^{2}}{2!}+\frac{\alpha^{4}}{4!}-\frac{\alpha^{6}}{6!}+\cdots \frac{{\left(-1\right)}^{n}\alpha^{2n}}{2n!}+\frac{{\left(-1\right)}^{n+1}\cos\xi }{(2n+2)!}\alpha^{2n+2},\;\xi \in \left(0,\pi \right).$$

Let $\alpha =\frac{2\pi e_{u}}{q}$, we have $\cos\frac{2\pi e_{u}}{q}\in \left[-1,1\right]$. We find that starting from the third term, the positive term is greater than the negative term in two adjacent terms, (i.e., when n≥1 and *n* is even, $\frac{1}{2n!}{\left(\frac{2\pi e_{u}}{q}\right)}^{2n}-\frac{\cos\xi }{(2n+2)!}{\left(\frac{2\pi e_{u}}{q}\right)}^{2n+2}>0$; when n≥2 and *n* is odd, $\frac{1}{(2n-2)!}{\left(\frac{2\pi e_{u}}{q}\right)}^{2n-2}-\frac{1}{2n!}{\left(\frac{2\pi e_{u}}{q}\right)}^{2n}>0$).

So, we have $\cos\frac{2\pi e_{u}}{q}\geq 1-\frac{1}{2}{\left(\frac{2\pi e_{u}}{q}\right)}^{2}$. Then

(6) $$E\left(\cos\frac{2\pi e_{u}}{q}\right)\geq E\left(1-\frac{1}{2}{\left(\frac{2\pi e_{u}}{q}\right)}^{2}\right)=1-\frac{2\pi^{2}}{q^{2}}E\left(e_{u}^{2}\right)=1-\frac{2\pi^{2}}{q^{2}}\cdot \sigma^{2}$$

For any 0<δ<1, by using Hoeffding bound, we can obtain

(7) $$\begin{array}{cc} \; & \mathrm{Pr}[\sum_{u\in Z_{q}^{n}}\left(\cos\frac{2\pi e_{u}}{q}-E\left(1-\frac{1}{2}{\left(\frac{2\pi e_{u}}{q}\right)}^{2}\right)\right)\leq -\delta q^{n}]\\ \; & =\mathrm{Pr}\left[\sum_{u\in Z_{q}^{n}}\left(\cos\frac{2\pi e_{u}}{q}\right)\leq \left(1-\frac{2\pi^{2}}{q^{2}}\cdot \sigma^{2}\right)-\delta \right)q^{n}]\\ \; & \leq \mathrm{Pr}[\sum_{u\in Z_{q}^{n}}\left(\cos\frac{2\pi e_{u}}{q}-E\left(\cos\left(\frac{2\pi e_{u}}{q}\right)\right)\leq -\delta q^{n}\right]\\ \; & <e^{-2\delta^{2}q^{2n}/4}, \end{array}$$

Using (6) and (7), we have

(8) $$\sum_{u\in Z_{q}^{n}}\cos\frac{2\pi e_{u}}{q}\geq \sum_{u\in Z_{q}^{n}}E(\cos\left(\frac{2\pi e_{u}}{q}\right)-\delta q^{n}\geq \left(1-\frac{2\pi^{2}}{q^{2}}\cdot \sigma^{2}-\delta \right)q^{n}$$

Since $\cos\frac{2\pi e_{u}}{q}\in \left[-1,1\right]$, for any 0<δ<1, using (4), the probability of outputting sk is

(9) $$P\geq \frac{1}{q^{2n}}{\left(\left(1-\frac{2\pi^{2}}{q^{2}}\cdot \sigma^{2}-\delta \right)q^{n}\right)}^{2}={\left(1-\frac{2\pi^{2}}{q^{2}}\sigma^{2}-\delta \right)}^{2}$$

## 4. Quantum Misuse Attack

In this section, we first give a KR-CCA attack based on an improved quantum algorithm for solving quantum LWE. Then, we discuss the security of Frodo. In this attack, we consider an adversary with quantum access to a decryption oracle.

We consider the meta-PKC construction in Section 2.2, let $S_{sk}=Z_{q}^{n_{sk}},S_{A}=Z_{q}^{n_{A}},S_{B}=Z_{q}^{n_{B}},S_{t}=Z_{q}^{n_{t}},S_{U}=Z_{q}^{n_{U}},S_{V}=Z_{q}^{n_{V}}$. Define $W_{U}=V-U\times sk,pt^{\prime }=decode\left(W_{U}\right),Z_{U}=V-encode\left(pt^{\prime }\right)$, where $U\in S_{U},V\in S_{V}$. Hence, for any *V*

(10) $$\begin{array}{cc} \; & Z_{U}=V-encode\left(pt^{\prime }\right)\\ \; & =V-encode(decode(V-U\times sk))\\ \; & =V-\left(V-U\times sk\right)+\delta_{U})\\ \; & =U\times sk+\delta_{U}, \end{array}$$

$\delta_{U}$ denotes the error introduced by encoding/decoding and $\delta_{U}$ follows the uniform distribution. Then, the decryption oracle can make the following mapping:

$$|U\;V\;Z\rangle \to |U\;V\;Z+Z_{U}\rangle$$

In Table 2, the decryption algorithm returns plaintext $pt^{\prime }$, so the $Z_{U}$ can be obtained.

### 4.1. Key Recovery Algorithm

Define $S_{sk}=S_{B}=Z_{q}^{nm},S_{A}=Z_{q}^{n^{2}},S_{t}=S_{U}=Z_{q}^{mn},S_{V}=Z_{q}^{m^{2}}$. The bilinear mappings are matrix multiplications; let

$$U={\left(\begin{array}{c} U_{0}\\ U_{1}\\ \cdots \\ U_{m-1} \end{array}\right)}_{m\times n},sk={\left(\begin{array}{cccc} sk_{0} & sk_{1} & \cdots & sk_{m-1} \end{array}\right)}_{n\times m}$$

For $i\in \left[m\right],U_{i}\in Z_{q}^{n}$ is the *i*th row of *U*, and for $j\in \left[m\right],sk_{j}\in Z_{q}^{n}$ is the *j*th column of sk.

In the following, we give the quantum key recovery attack algorithm based on LWE encryption schemes in Algorithm 2. This algorithm can recover the key with constant success probability.
**Algorithm 2:** Quantum key recovery attack.Input: i,j∈[m] and *V*Quantum oracle: $|U\;\;V\;Z\rangle \to |U\;V\;Z+Z_{U}\rangle$   1: Set the quantum state to $|0\;V\;{\left(1_{ij}\right)}_{i=j}\rangle \in Z_{q}^{mn}\times Z_{q}^{m^{2}}\times Z_{q}^{m^{2}}$.   2: Make a quantum Fourier transform on the first and third registers.   3: Make a quantum oracle query and obtain (by writing $Z^{\prime }=Z+Z_{U}$).      $\frac{1}{\sqrt{q^{mn}}}\frac{1}{\sqrt{q^{m^{2}}}}\sum_{U,Z^{\prime },}\left(\prod_{i=j}\omega^{Z_{ij}^{\prime }-Z_{U_{ij}}}\right)|U\;V\;Z^{\prime }\rangle$.   4: Discard the last two registers and apply the quantum Fourier transform.   5: Measure the first register and output α.

**Theorem** **1.** *Let $U\in Z_{q}^{mn}$, $Z_{U_{ij}}={\left(U\times sk\right)}_{ij}+\delta_{U_{ij}}$, let the expectation value of the error $\delta_{U_{ij}}$ be μ and the variance of the error $\delta_{U_{ij}}$ be $\sigma^{2}$. Then, the algorithm of Algorithm 2 can recover the full key sk with constant probability β.*

**Proof.**  Prepare the state $|0\;V\;{\left(1_{ij}\right)}_{i=j}\rangle \in Z_{q}^{mn}\times Z_{q}^{m^{2}}\times Z_{q}^{m^{2}}$. By making a quantum Fourier transform on the first and third registers, we obtain

$$\frac{1}{\sqrt{q^{mn}}}\frac{1}{\sqrt{q^{m^{2}}}}\sum_{U,Z}\left(\prod_{i=j}\omega^{Z_{ij}}\right)|U\;V\;Z\rangle .$$

After querying a quantum oracle and letting $Z^{\prime }=Z+Z_{U}$, we have

$$\frac{1}{\sqrt{q^{mn}}}\frac{1}{\sqrt{q^{m^{2}}}}\sum_{U,Z^{\prime },}\left(\prod_{i=j}\omega^{Z_{ij}^{\prime }-Z_{U_{ij}}}\right)|U\;V\;Z^{\prime }\rangle .$$

If we discard the last two registers and apply quantum Fourier transform, we obtain

$$\frac{1}{q^{mn}}\sum_{U,\alpha }\left(\prod_{i=j}\omega^{-Z_{U_{ij}}}\right)\omega^{U\cdot \alpha }|\alpha \rangle .$$

Then, we perform a complete measurement in the computational basis. The probability of obtaining Pr[α] is given by

(11) $$\begin{array}{cc} \; & Pr\left[\alpha \right]=∥\frac{1}{q^{mn}}\sum_{U}\left(\prod_{i=j}\omega^{-Z_{U_{ij}}}\right)\omega^{U\cdot \alpha }{∥}^{2}\\ \; & =∥\frac{1}{q^{mn}}\sum_{U}\left(\prod_{i=j}\omega^{-U_{i}\cdot sk_{j}-\delta_{U_{ij}}}\right)\left(\prod_{i=j}\omega^{U_{i}\cdot sk_{j}}\right){∥}^{2}\\ \; & =∥\frac{1}{q^{mn}}\sum_{U_{ij}}\left(\prod_{i=j}\omega^{-\delta_{U_{ij}}}\right){∥}^{2}\\ \; & \geq {\left(\frac{1}{q^{2mn}}{\left(\sum_{U_{ij}}Re\left(\omega^{-\delta_{U_{ij}}}\right)\right)}^{2}\right)}^{m}, \end{array}$$

where α is a matrix of *m* blocks, and the size of each block is *n* for α such that $U_{i}\cdot \alpha_{j}=0$ (i.e., $\alpha_{j}=0$) for i≠j and $\alpha_{j}=sk_{j}$ for i=j.

Using (9), we obtain

(12) $$Pr\left[\alpha \right]\geq {\left(1-\frac{2\pi^{2}}{q^{2}}\left(\mu^{2}+\sigma^{2}\right)-\delta \right)}^{2m}$$

We can further reduce the number of oracle calls with the same success probability. The specific analysis is as follows.

We can see that the success probability of obtaining one column of sk is $p={\left(1-\frac{2\pi^{2}}{q^{2}}\left(\mu^{2}+\sigma^{2}\right)-\delta \right)}^{2}$. Suppose we can fully recover sk with constant probability Pr[α]=β by *k* queries. Then, the probability of recovering the first column of sk at least once in *k* queries is $1-{\left(1-p\right)}^{k}$. So, we can fully recover secret sk with probability ${\left(1-{\left(1-p\right)}^{k}\right)}^{m}$. We expect

(13) $${\left(1-{\left(1-p\right)}^{k}\right)}^{m}\geq \beta ,$$

and then we can obtain the value of *k*. We will analyze it in detail in the following Section 4.2, using Frodo as the example. □

### 4.2. Application to Post-Quantum Cryptosystem Frodo

We consider the IND-CPA secure public key encryption scheme FrodoPKE, which is based on the public-key encryption scheme presented by Lindner and Peikert in [24]. FrodoPKE is a family of conservative yet practical post-quantum public key encryptions with security based on the hardness of the LWE problem.

Before giving the public-key encryption scheme of Frodo, we first describe how bit strings are encoded as mod-q integer matrices. Let *D* denote the number of bits used for encoding. The encoding function ec(·) encodes an integer $0\leq pt<2^{D}$ as an element in $Z_{q}$ by multiplying it by $\frac{q}{2^{D}}$:

(14) $$ec\left(pt\right):=pt\cdot \frac{q}{2^{D}}.$$

By applying ec(·) to *D*-bit sub-strings sequentially and filling the matrix row by row entry-wise, the function Frodo.Encode encodes bit strings of length $l=D\cdot m\cdot \bar{n}$ as $m\cdot \bar{n}$ matrices with entries in $Z_{q}$ in left column of Table 3. The corresponding decoding function Frodo.Decode is defined as shown in right column of Table 3. It decodes the $m\cdot \bar{n}$ matrix *M* into a bit string of length $l=D\cdot m\cdot \bar{n}$ and extracts *B* bits from each entry by applying the function de(c):

(15) $$de\left(c\right):=\left\lfloor c\cdot \frac{2^{D}}{q}\right\rfloor \mathrm{mod}\;2^{D}.$$

Let $m,n,\bar{n}$ be integer parameters and q≥2 be an integer power of 2. In Table 4, we depict the public-key encryption scheme of Frodo. The symbol $\overset{\chi }{\leftarrow }$ denotes a sample is chosen according to χ. FrodoPKE works with $S_{sk}=S_{B}=Z_{q}^{n\bar{n}},S_{A}=Z_{q}^{n^{2}},S_{t}=S_{U}=Z_{q}^{mn}$, and $S_{V}=Z_{q}^{m\bar{n}}$ with $L_{\infty }$ norm, $\delta_{U}\in \left[-\rho_{+},\rho_{+}\right]$, where $\rho_{+}=\frac{q}{8}$, $M=encode\left({\mathrm{pt}}^{\prime }\right)\in Z_{q}^{m\times \bar{n}}$.

In FrodoPKE, χ is a discrete Gaussian distribution, and the error $\delta_{U}$ introduced by encoding/decoding is chosen according to uniform distribution with range $\left[-\rho_{+},\rho_{+}\right]$. In Table 5, we give the other parameters of Frodo.

For Frodo640, $q=2^{15}$, $\delta_{U}$ is chosen according to uniform distribution with range $\left[-\rho_{+},\rho_{+}\right]$; this is $\left[-2^{12},2^{12}\right]$. The variance of $\delta_{U}$ is 5,593,770.67; then

$$Pr\left[sk_{0}\right]\geq {\left(1-\frac{2\pi^{2}}{q^{2}}\left(\mu^{2}+\sigma^{2}\right)-\delta \right)}^{2}=0.81$$

Using Equation (11), ${\left(1-{\left(1-0.81\right)}^{k}\right)}^{8}=0.81^{8}$, we can obtain k=1. So, we can fully recover the secret sk with probability more than $0.81^{8}=0.18$ with only 1 query.

For Frodo976, $q=2^{16}$, $\delta_{U}$ is chosen according to uniform distribution with range $\left[-\rho_{+},\rho_{+}\right]$, this is $\left[-2^{12},2^{12}\right]$. The variance of $\delta_{U}$ is 5593770.67; then

$$Pr\left[sk_{0}\right]\geq {\left(1-\frac{2\pi^{2}}{q^{2}}\left(\mu^{2}+\sigma^{2}\right)-\delta \right)}^{2}=0.95$$

Using Equation (11), ${\left(1-{\left(1-0.95\right)}^{k}\right)}^{8}=0.95^{8}$, we can obtain k=1. So, we can fully recover the secret sk with probability more than $0.95^{8}=0.66$ with only 1 query.

For Frodo1344, $q=2^{16}$, $\delta_{U}$ is chosen according to uniform distribution with range $\left[-\rho_{+},\rho_{+}\right]$; this is $\left[-2^{11},2^{11}\right]$. The variance of $\delta_{U}$ is 1,398,784; then

$$Pr\left[sk_{0}\right]\geq {\left(1-\frac{2\pi^{2}}{q^{2}}\left(\mu^{2}+\sigma^{2}\right)-\delta \right)}^{2}=0.99$$

Using Equation (11), ${\left(1-{\left(1-0.99\right)}^{k}\right)}^{8}=0.99^{8}$, we can obtain k=1. So, we can fully recover the secret sk with probability more than $0.99^{8}=0.92$ with only 1 query.

## 5. Conclusions and Discussion

In this paper, we developed a quantum algorithm to recover the key against LWE-based NIST candidates PKEs. Based on the improved quantum algorithm for solving LWE, we considered the success probability for solving the quantum LWE problem when the noise follows a discrete Gaussian distribution. Then, we proposed a new quantum key-recovery attack algorithm and gave a specific analysis for FrodoPKE. Compared with the existing algorithm [22], our algorithm can reduce the number of oracle calls with the same success probability.

In reality, the key is usually misused in a very short time, which leads to the number of queries being taken as the prime optimization goal with respect to misuse attack. During this short time, if an adversary can only make one oracle query, the misuse attack that requires four queries does not work for an adversary. However, our algorithm only needs one query to recover the key with probability 1. Therefore, the fewer oracle queries required, the greater the advantage for an adversary.

## Figures and Tables

**Table 1:** Three types of attacks on several lattice-based cryptosystems. P denotes the success probability, and O denotes the total number of oracle calls required to recover the full key with probability 1 by iterating the attack.

	GKZ-Based Quantum KR-CCA Attack [22]	AJOP-Based Quantum KR-CCA Attack [22]	Improved Quantum KR-CCA Attack
	P O	P O	P O
Frodo	$2^{-13}$ $2^{17}$	$2^{-2}$ $2^{2}$	$2^{-2}$ 1

**Table 2:** The meta-cryptosystem defined on the algebra.

Algorithm setup($1^{\lambda }$):	Algorithm enc(pp,pk,pt;coinB):
1: set up the algebra and define pp	1: parse pk=(A,B)
2: return pp	2: pick random sparse $t\in S_{t},e\in S_{U}$
	and $f\in S_{V}$ by using coinB
**Algorithm gen(pp;coinA):**	3: U=t×A+e
1: pick a random $A\in S_{A}$ and random sparse	4: V=t×B+f+encode(pt)
$sk\in S_{sk}$ and $d\in S_{B}$ by using coinA	5: return ct=(U,V)
2: B=A×sk+d	
3: pk=(A,B)	**Algorithm dec(pp,sk,ct):**
4: return (sk,pk)	1: parse ct=(U,V)
	2: W=V−U×sk
	3: $pt^{\prime }=decode\left(W\right)$
	4: return $pt^{\prime }$

**Table 3:** Encode and Decode Functions of Frodo.

Frodo.Encode	Frodo.Decode
input: bit string $\mathrm{pt}\in {\left\{0,1\right\}}^{l},l=D\cdot m\cdot \bar{n}$	input: matrix $M\in Z_{q}^{m\times \bar{n}}$
output: matrix $M\in Z_{q}^{m\times \bar{n}}$	output: bit string $\mathrm{pt}\in {\left\{0,1\right\}}^{l},l=D\cdot m\cdot \bar{n}$
	
1: for (i=0;i<m;i←i+1) do	1: for (i=0;i<m;i←i+1) do
2: for $\left(j=0;j<\bar{n};j\leftarrow j+1\right)$ do	2: for $\left(j=0;j<\bar{n};j\leftarrow j+1\right)$ do
3: $pt\leftarrow \sum_{l=0}^{D-1}{\mathrm{pt}}_{(i\cdot \bar{n}+j)D+l}\cdot 2^{l}$	3: $pt\leftarrow de\left(M_{i,j}\right)=\left\lfloor M_{i,j}\cdot \frac{2^{D}}{q}\right\rceil \mathrm{mod}2^{D}$
4: $M_{i,j}\leftarrow ec\left(pt\right)=pt\cdot \frac{q}{2^{D}}$	4: $pt=\sum_{l=0}^{D-1}pt_{l}\cdot 2^{l}\;$ where $pt_{l}\in \left\{0,1\right\}$
5: return $M={\left(M_{i,j}\right)}_{0\leq i<m,0\leq j<\bar{n}}$	5: for (l=0;l<D;l←l+1) do
	6: ${\mathrm{pt}}_{(i\cdot \bar{n}+j)\cdot D+l}\leftarrow pt_{l}$
	7: return pt

**Table 4:** The CPA version of Frodo.

Alice		Bob
1. Frodo.CPAPKE.Gen()		
1.1 Generate matrix $A\in Z_{q}^{n\times n}$		
1.2 Sample $S,E\overset{\chi }{\leftarrow }Z_{q}^{n\times \bar{n}}$		2. $\mathrm{pt}\in {\left\{0,1\right\}}^{lm\bar{n}}$
1.3 B=A·S+E		3. Frodo.CPAPKE.Enc(B,m)
1.4 Output (B,S)	$\overset{B}{⟶}$	3.1 Generate matrix $A\in Z_{q}^{n\times n}$
		3.2 $S^{\prime },E^{\prime }\overset{\chi }{\leftarrow }Z_{q}^{m\times n}$, $E^{\prime \prime }\overset{\chi }{\leftarrow }Z_{q}^{m\times \bar{n}}$
		3.3 $U=S^{\prime }A+E^{\prime }$
4. Frodo.CPAPKE.Dec(U,V,S)		3.4 $V=S^{\prime }B+E^{\prime \prime }+encode\left(\mathrm{pt}\right)$
4.1 M=V−US	$\overset{U,V}{⟵}$	3.5 Output (U,V)
4.2 ${\mathrm{pt}}^{\prime }=decode\left(M\right)$		
4.3 Output $pt^{\prime }$		

**Table 5:** Parameter sets for Frodo.

	*n*	*q*	*D*	$m\times \bar{n}$	sk Ranges	$\rho_{+}$
Frodo640	640	$2^{15}$	2	8×8	[−12,12]	$2^{12}$
Frodo976	976	$2^{16}$	3	8×8	[−10,10]	$2^{12}$
Frodo1344	1344	$2^{16}$	4	8×8	[−6,6]	$2^{11}$

## Data Availability

The data presented in this study are available within the article.

## Abbreviations

PKE	Public-key encryption
KEM	Key encapsulation mechanisms
NIST	National Institute of Standards and Technology
PQC	Post-quantum cryptography
LWE	Learning with error
PKC	Public key cryptosystem
KR-PCA	Key recovery under plaintext checking attacks
KR-CPA	Key recovery under chosen plaintext attacks
KR-CC	Key recovery under chosen ciphertext attacks
IND-CPA	INDistinguishability against chosen plaintext attacks
IND-CCA	INDistinguishability against chosen ciphertext attacks
